# Natural Vertical Transmission of Zika Virus in Larval *Aedes aegypti* Populations, Morelos, Mexico

**DOI:** 10.3201/eid2508.181533

**Published:** 2019-08

**Authors:** Mónica Izquierdo-Suzán, Selene Zárate, Jesús Torres-Flores, Fabián Correa-Morales, Cassandra González-Acosta, Edgar E. Sevilla-Reyes, Rosalia Lira, Sofía L. Alcaraz-Estrada, Martha Yocupicio-Monroy

**Affiliations:** Universidad Autónoma de la Ciudad de Mexico, Mexico City, Mexico (M. Izquierdo-Suzán, S. Zárate, M. Yocupicio-Monroy);; Instituto Politécnico Nacional, Mexico City (J. Torres-Flores);; Centro Nacional de Programas Preventivos y Control de Enfermedades, Mexico City (F. Correa-Morales, C. González-Acosta);; Instituto Nacional de Enfermedades Respiratorias, Mexico City (E.E. Sevilla-Reyes);; Instituto Mexicano del Seguro Social, Mexico City (R. Lira);; Instituto de Seguridad y Servicios Sociales de los Trabajadores del Estado, Mexico City (S.L. Alcaraz-Estrada)

**Keywords:** Zika virus, Aedes aegypti, natural vertical transmission, larvae, Mexico, viruses, mosquitoes, vector-borne infections

## Abstract

We characterized natural vertical transmission of Zika virus in pools of *Aedes aegypti* larvae hatched from eggs collected in Jojutla, Morelos, Mexico. Of the 151 pools analyzed, 17 tested positive for Zika virus RNA; infectious Zika virus was successfully isolated from 1 of the larvae pools (31N) in C6/36 cells. Real-time quantitative PCR and indirect immunofluorescence assays confirmed the identity of the isolate, named Zika virus isolate 31N; plaque assays in Vero cells demonstrated the isolate’s infectivity in a mammalian cell line. We obtained the complete genome of Zika virus isolate 31N by next-generation sequencing and identified 3 single-nucleotide variants specific to Zika virus isolate 31N using the meta-CATS tool. These results demonstrate the occurrence of natural vertical transmission of Zika virus in wild *Ae. aegypti* mosquitoes and suggest that this transmission mode could aid in the spread and maintenance of Zika virus in nature.

Zika virus is an enveloped, positive-sense, single-stranded sRNA, arthropod-borne virus (arbovirus) that is classified in the genus *Flavivirus*, family *Flaviviridae*. Zika virus is closely related to other viruses of medical importance, such as dengue, West Nile, and yellow fever viruses (*1*). Zika virus was discovered in Uganda in 1947, but it has attracted the attention of specialists in the past few years because of its rapid spread through the Pacific and into the Americas in 2015, as well as the severe neurologic manifestations associated with Zika virus infections, such as neonatal microcephaly and Guillain-Barré syndrome (*2*).

Several studies carried out under laboratory conditions have demonstrated that Zika virus can infect many different *Aedes* mosquito species (*3*); still, the key species for the transmission of Zika virus to humans are *Ae. aegypti* and *Ae. albopictus* (*4*–*6*). In this study, we focused on the species *Ae. aegypti*, which has an urban behavior and is usually in close contact with humans (*7*). To date, 2 different mechanisms by which *Ae. aegypti* mosquitoes can become infected with flaviviruses have been described: horizontal transmission and vertical transmission (*8*).

Horizontal transmission is considered the most common mode of transmission of arboviruses between mosquitoes and their vertebrate hosts and is responsible for the maintenance of arboviruses in nature, particularly during disease outbreaks. In contrast, the environmental maintenance of dengue virus (DENV) during interepidemic periods is thought to be caused by vertical transmission of the virus from the infected adult mosquitoes to their offspring for >7 successive generations (*9*–*11*). Both mechanisms together are thought to be essential for the survival of viral pathogens in their habitats, preventing their extinction during harsh environmental conditions or in populations in which the presence of susceptible mammal hosts is low.

Vertical transmission of Zika virus in *Ae. aegypti* mosquitoes has been evaluated under laboratory conditions by searching for the presence of Zika virus RNA in several organs from the offspring of infected mosquitoes, demonstrating the presence of viral RNA in the guts and salivary glands of the offspring (*12*). Additional studies have also demonstrated the presence of infectious Zika virus in the offspring of artificially infected *Ae. aegypti* and *Ae. albopictus* mosquitoes, suggesting that vertical transmission can occur in laboratory-bred mosquitoes (*13*). However, evidence is insufficient to confirm that vertical transmission is a principal maintenance mechanism for Zika virus in wild *Aedes* mosquitoes.

In this study, we sought to demonstrate natural vertical transmission in *Ae. aegypti* mosquitoes by detecting viral RNA and isolating infectious Zika virus from larvae hatched from field-collected eggs. We also assessed the infectivity of the isolate in a mammalian cell line and obtained the complete genome of the virus by next-generation sequencing (NGS).

## Materials and Methods

### Study Site

We collected mosquito egg samples in the municipality of Jojutla, located in the southern region of the state of Morelos, Mexico (18°36′N, 99°10′W). Arboviral infections are common in this municipality, which is usually hot and dry during October–April and warm and humid during May–September. During 2016, the incidence of Zika virus infections increased in Morelos; 269 cases were reported by the end of the year, of which 69 cases were from Jojutla. The reported cases in Jojutla were recorded as follows: 1 in August, 17 in September, 20 in October, 25 in November, and 6 in December (*14*).

### Egg Collection and Hatching

**Figure 1:**
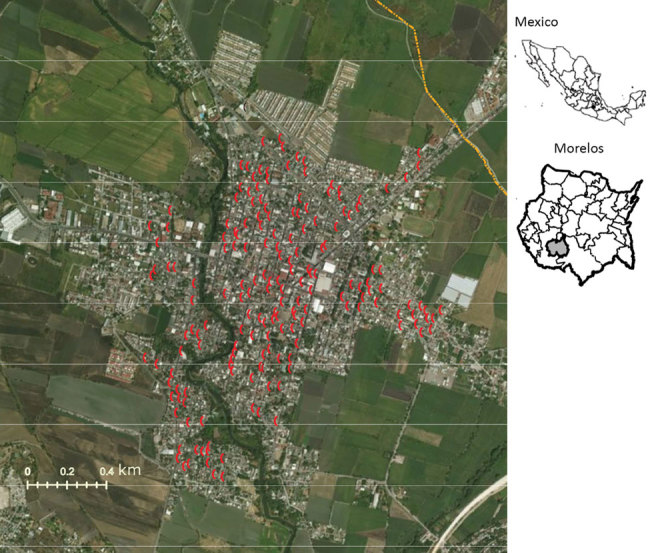
Location of ovitraps in the municipality of Jojutla, Morelos, Mexico (red). The ovitraps were set according to the guidelines of the Vector Transmitted Diseases Program of the National Center of Preventive Programs and Disease Control (CENAPRECE; http://www.cenaprece.salud.gob.mx/programas/interior/portada_vectores.html). Insets show location of Morelos in Mexico (top) and Jojutla in Morelos (bottom).

We collected *Ae. aegypti* eggs in collaboration with the National Center of Preventive Programs and Disease Control (CENAPRECE), following regulations for entomological surveillance with ovitraps (NOM-032-SSA2–2014). In 2016, 180 ovitraps were placed and maintained throughout the year in different locations in Jojutla (Figure 1). We determined the locations of the ovitraps on the basis of the incidence of human arbovirus infections. We collected the eggs using filter paper, which we then air dried for 2 days and stored in paper bags for further use. 

We placed the egg papers in water containers and incubated them at 37°C for 1 wk under 24-h light/dark cycles. After the incubation period, we collected larvae in stages 2–3 and separated them into pools of 20–30 larvae. We macerated each of the pools in viral transport media (5% BSA, 100 U/mL penicillin, 100 μg/mL streptomycin, 2.5 μg/mL fungizone, and NaHCO_3_ in Hank’s solution) using a pestle mixer (Thomas Scientific, https://www.thomassci.com) and stored pools at −80°C for further analysis.

### Detection of Zika Virus in Larvae of *Aedes aegypti*

We incubated the macerated larvae pools with Trizol (Invitrogen, https://www.thermofisher.com) for 10 min, extracted total RNA from the lysates using Zymo-Spin RNA extraction columns (Zymo Research, https://www.zymoresearch.com), and quantified the RNA with a Nano Drop 2000 (Thermo Scientific, https://www.thermofisher.com). We detected the presence of Zika virus RNA by quantitative reverse transcription PCR (qRT-PCR) using the TaqMan system (https://www.thermofisher.com*)* with primers Zikv 1086 Fw 5′-CCGCTGCCCAACACAAG-3′ and Zikv 1162c Rv 5′-CCACTAACGTTCTTTTGCAGACAT-3′; and probe Zikv 1107-FAM probe 5′-AGCCTACCTTGACAAGCAGTCAGACACTCAA-3′, as previously described by Lanciotti et al. (*15*). We calculated the minimum infection rate (MIR) of the larvae pools by dividing the number of positive larvae pools by the total number of larvae tested and multiplying by 1,000.

### Viral Isolation and Identification

We selected the larvae pools that tested positive for Zika virus RNA and displayed the lowest quantitation cycle (Cq) values (range 22.3–33.4) for viral isolation. In brief, we diluted macerated larvae pools in maintenance medium (EMEM medium with 1% FBS, 100 U/mL penicillin, and 100 μg/mL streptomycin) and filtered them through 0.22 μm membranes (Millex, http://www.emdmillipore.com). Twenty-four hours before infection, we seeded C6/36 cells in 24-well plates at 80% of confluence in growth medium (EMEM medium supplemented with 10% FBS, 100 U/mL penicillin, and 100 μg/mL streptomycin) and incubated them at 28°C in a 5% CO_2_ atmosphere. Cells were adsorbed with the clarified larvae macerates for 2 h at 28°C. After the incubation period, we added fresh maintenance medium to the cells and left the infection to proceed for 7 d at 28°C and a 5% CO_2_ atmosphere until a cytopathic effect was observable. We tested the supernatants of the infected cells for the presence of Zika virus RNA by qRT-PCR, as described earlier in this section. We performed 4 passages of each isolate in C6/36 cells to increase viral titers for NGS.

### Indirect Immunofluorescence Assay

For the indirect immunofluorescence assays, we grew Vero cells over glass coverslips and then infected them with the Zika virus isolate. At 48 hours postinfection, we fixed cells with 4% paraformaldehyde and then incubated them with ice-cold methanol for 10 min. We blocked the cells using 10% FBS in phosphate-buffered saline (PBS) for 1 h and then incubated them overnight at 4°C with a 1:500 dilution of the monoclonal antibody 4G2. After the incubation period, we washed the cells twice with PBS and then incubated them with an Alexa Fluor 488-conjugated goat antimouse IgG antibody (Jackson Immunoresearch, https://www.jacksonimmuno.com) for 1 h. We washed the cells twice with PBS and then mounted them with VECTASHIELD medium with DAPI (Vector Laboratories, https://vectorlabs.com) for confocal microscopy (Nikon, https://www.nikon.com).

### Plaque Assays

For the plaque assays, we seeded Vero cells in 24-well plates until they reached a confluence of 80%. We used 10-fold serial dilutions of the Zika virus isolate to infect the cell monolayers and left them to adsorb for 1 h. After the adsorption period, we removed the virus and overlaid cell monolayers with 1 mL of DMEM (Invitrogen) supplemented with 2% carboxymethyl-cellulose (Sigma-Aldrich, https://www.sigmaaldrich.com), 2% FBS, 100 U/mL penicillin, 100 μg/mL streptomycin, and 2 mM L-glutamine (Gibco, *https://*www.thermofisher.com) and incubated at 37°C for 4 d. We fixed the cells with 4% paraformaldehyde in PBS and counterstained them with crystal violet-formaldehyde (Sigma-Aldrich).

### Full Genome Sequencing and Assembly

For the complete genome sequencing of the Zika virus isolate, we depleted 200 ng of total RNA extracted from the supernatants of infected Vero cell monolayers of rRNA using the NEBNext rRNA Depletion Kit (human/mouse/rat) following the manufacturer’s instructions (New England Biolabs*,* https://www.neb.com). We constructed RNaseq Illumina shotgun libraries at the Unidad Universitaria de Secuenciación Masiva y Bioinformática-Instituto de Biotecnología and sequenced them using paired-end sequencing with a MySeq system (Illumina, https://www.illumina.com). We assessed the quality of reads using FASTQC (http://www.bioinformatics.babraham.ac.uk/projects/fastqc); low-quality positions and reads were eliminated using in-house scripts. We assembled the valid reads without reference using the program Trinity 2.8 (*16*) and evaluated the assembly using Qualimap (*17*). Finally, we verified the identity of the contig using blastn (http://blast.ncbi.nlm.nih.gov/Blast.cgi).

### Phylogenetic Reconstruction

To determine the phylogenetic relatedness of the Zika virus isolate, we retrieved relevant Zika virus open reading frame (ORF) RNA sequences from the Virus Pathogen Resource (ViPR) database (*18*) (http://www.viprbrc.org). We removed identical sequences and those with undetermined bases from alignment. We applied near-identity clustering (0.999) to the remaining sequences in Cd-hit-test (*19*) and used 98 sequences for phylogenetic inference by maximum likelihood in RAxML (*20*) under a general time reversible plus gamma substitution model. We reconstructed the tree with 200 bootstrap replicas. We normalized branch lengths and condensed nodes with bootstrap values <50% to emphasize tree topology.

### Variant Analysis

To determine the presence of variants in the larva-derived sequence, we used the alignment to identify positions that had mutations unique to our sequence or that were primarily shared with other mosquito-derived sequences and that were absent or rare in the human-derived genomes. To this end, we examined the alignment with the tool meta-CATS (*21*), which performs a χ^2^ test to find positions with different polymorphism distribution between groups.

## Results

### Zika Virus Isolation and Identification in *Ae. aegypti* Larvae

During the study period, we analyzed 151 larvae pools by qRT-PCR in search of Zika virus RNA. Only 17 (10.8%) of the pools tested positive: 9 (5.7%) from the larvae raised from the eggs collected in June and 8 (5.1%) from larvae raised from the eggs collected in November. To determine the proportion of infected larvae in the population, we calculated MIRs for the 2 collection periods (June and November 2016) and found an increase in the MIR observed from the larvae raised from the eggs collected in June (2.5) to the MIR from the eggs collected in November (6.9) (Table 1).

**Table 1:** MIR for Zika virus in *Aedes aegypti* larvae, Jojutla, Morelos, Mexico, June and November 2016*

Collection date	Positive pools/ analyzed pools	Specimens analyzed	MIR
June	9/105	3150	2.8
November	8/46	1150	6.9

*MIR, minimum infection rate.

**Figure 2:**
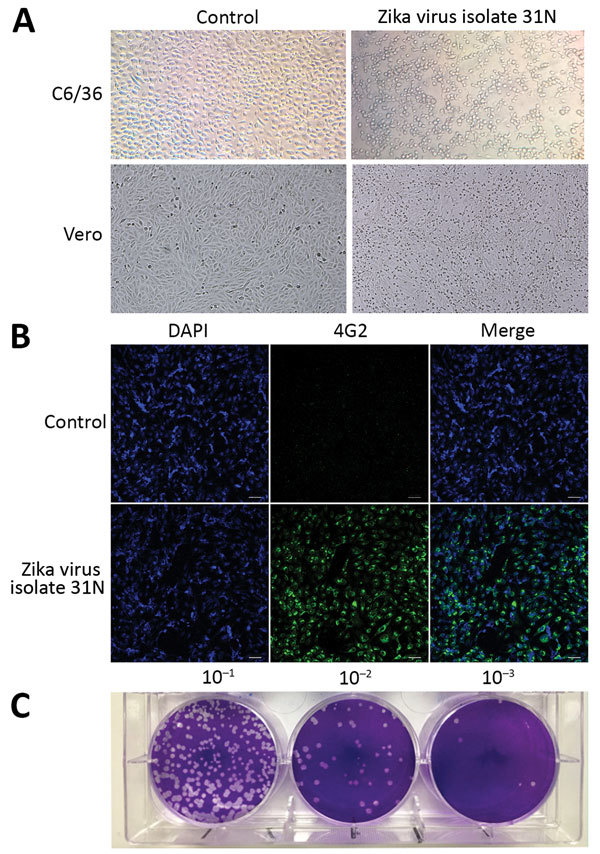
Phenotypic analysis of Zika virus isolate 31N from an *Aedes aegypti* larval pool, Jojutla, Morelos, Mexico. A) Cytopathic effect of the Zika virus isolate 31N in C6/36 and Vero cells. The left panel shows mock infected cells. Original magnification ×20. B) Infected Vero cells with Zika virus isolate 31N at a multiplicity of infection of 0.1 and mock infected cells. Nuclei are stained in blue (DAPI), and the envelope protein is stained in green (4G2). Original magnification ×20. C) Plaque assay of Zika virus isolate 31N in Vero cells. Serial decimal dilutions of Zika virus isolate 31N are depicted.

To confirm the presence of infectious Zika virus in the larvae pools, we used 11 of the 17 pools that tested positive for Zika virus RNA that displayed the lowest Cq values to attempt viral isolation. We were able to isolate infectious Zika virus from only 1 of the larvae pools (31N), as determined by the presence of cytopathic effect in C6/36 cells characterized by monolayer detachment (Figure 2, panel A) and the detection of Zika virus RNA in culture supernatants by qRT-PCR with a Cq value of 23.73.

The ability of the 31N isolate to infect a mammalian cell line was confirmed by the appearance of the characteristic cytopathic effect of Zika virus in infected Vero cell cultures, characterized by cell rounding and detachment (Figure 2, panel A). Moreover, immunofluorescent detection of the viral envelope (E) protein using the monoclonal antibody 4G2 revealed perinuclear staining (Figure 2, panel B). The plaque assays carried out in Vero cells revealed that the 31N isolate has the ability to produce lytic plaques; thus, this isolate can be considered cytopathic in mammalian cell culture (Figure 2, panel C).

### Complete Genome Sequencing of Zika Virus Isolate 31N

We isolated viral RNA from the supernatant of Vero cells infected with the fourth passage of Zika virus isolate 31N and processed it by RNA sequencing by the NGS MiSeq Illumina protocol. Around 40% of the total reads (6,535,816) were assembled in a single contig of 10,795 nt in length, with a mean depth of 42,000 reads. BLAST results of the assembled contig revealed that the strain ZIKV/Aedes.sp/MEX/MEX_I-7/2016, which belongs to a Zika virus isolated from *Aedes* mosquitoes obtained in the state of Chiapas, Mexico, had the highest identity (98.8%) with our isolate.

### Variant Analysis

We identified single-nucleotide variants (SNVs) in the Zika virus isolate 31N genome by aligning the sequence with other mosquito- and human-derived sequences obtained from the ViPR database and running the meta-CATS analytic tool, as described in the Materials and Methods section. To carry out this analysis, we grouped the sequences by the host of origin. We identified SNVs in positions 3176, 3286, and 5636 that were unique to the larva genome. On the other hand, the SNVs in positions 2071 and 3333 were found in human-derived sequences but were not present in other mosquito genomes. The SNV in position 2071 was shared only with a sequence from French Polynesia, likely an example of parallel evolution, whereas the polymorphism in position 3333 was common with 2 sequences from Mexico and may correspond to a local variant. Finally, we found 8 SNVs that were also present in other human and mosquito sequences but were more common in mosquitoes. Only 1 SNV was found in the E gene, whereas the rest were found in nonstructural (NS) genes: 5 in NS2A, 4 in NS3, 2 in NS5, and 1 in NS1 (Table 2).

**Table 2:** Residue diversity between Zika virus isolate 31N from an *Aedes aegypti* larval pool and human- and mosquito-derived genomes, Jojutla, Morelos, Mexico*

Position	Genome region	Human-derived sequences				Mosquito-derived sequence		p value
		SNVs, %	Origin of minority variant			SNVs, %	Origin of minority variant	
1008	E	T→C, 3.65	Mexico, United States, Thailand			T→C, 17.64	31N, Mexico	0.000253
2071†	NS1	C→T, 0.26	French Polynesia			C→T, 2.94	31N	0.03
2871	NS2A	T→C, 4.43	Mexico, United States			T→C, 35.29	31N, Mexico, United States	<0.0001
3176‡	NS2A	CS				A→C, 2.94	31N	0.01022
3286‡	NS2A	CS				A→G, 2.94	31N	0.01022
3333†	NS2A	A→G, 0.52	Mexico			A→G, 2.94	31N	>0.05
3788	NS2A	C→T, 0.26	Brazil			C→T, 8.82	31N, Mexico	<0.0001
4500	NS2A	A→G, 3.65	Mexico, United States, Philippines, South Korea			A→G, 17.64	31N, Mexico	0.003847
4624	NS3	G→A, 3.39	United States, Mexico			G→A, 17.64	31N, Mexico	0.002194
4980	NS3	T→C, 6.00	Mexico, Puerto Rico, Colombia, United States			T→C, 17.64	31N, Mexico	0.01
5636‡	NS3	CS				C→T, 2.94	31N	0.01022
7200	NS5	T→A 0.26; T→C, 3.91§		Mexico, USA, Suriname		T→C, 17.64	31N, Mexico	0.006182
9139	NS5	C→T, 0.52	Mexico			C→T, 11.76	31N, Mexico	<0.0001

*CS, conserved site in human-derived sequences; SNV, single nucleotide variant. †SNVs in human-derived sequences but not in other mosquito genomes.  ‡SNVs unique to the larva genome. §Two SNVs located in the same genome position.

### Phylogenetic Reconstruction of Zika Virus

**Figure 3:**
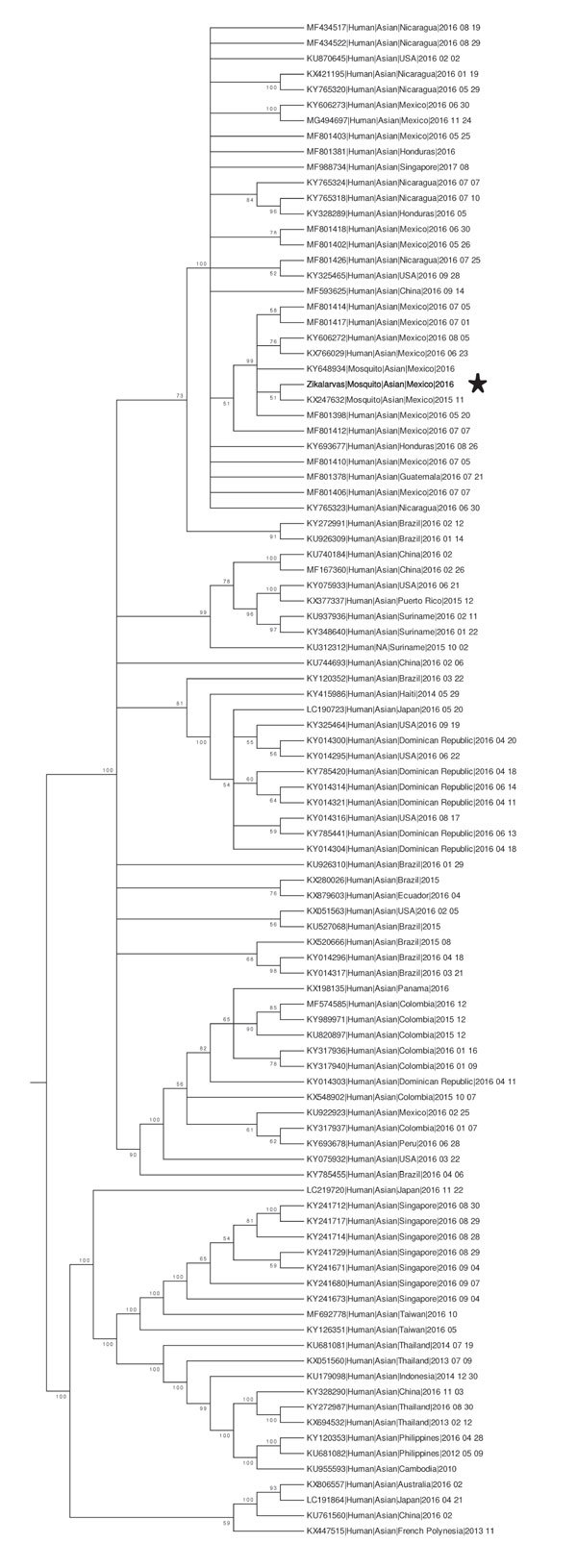
Condensed phylogenetic reconstruction of selected complete Zika virus genomes. This tree depicts the phylogenetic relationships between Zika virus isolate 31N from an *Aedes aegypti* larval pool, Jojutla, Morelos, Mexico (bold), and 98 complete genome sequences of Zika viruses obtained from the Virus Pathogen Resource database. GenBank accession numbers are provided. Nodes with bootstrap values support <50% were condensed and branch lengths were normalized to emphasize tree topology.

To characterize the evolutionary relationship between the 31N isolate and other Zika virus genomes that have been previously reported, we performed a phylogenetic analysis. The phylogenetic reconstruction of the complete genomes of 98 Zika virus sequences, including the 31N isolate, revealed that the 31N isolate belongs to the Asian-American lineage of Zika virus and clusters together with other sequences of human and mosquito origin from Mexico (Figure 3).

## Discussion

Natural vertical transmission of mosquito-carried viruses has been proposed as one of the main ecologic processes involved in the maintenance of these viruses in susceptible mosquito populations, particularly during interepidemic periods and harsh climate conditions when horizontal transmission becomes difficult (*11*,*22*). In Mexico, most of the studies regarding natural vertical transmission of arboviruses in mosquitoes have been carried out using DENV as a study model (*23*–*25*), so the diversity of viruses that can be transmitted from infected adult mosquitoes to their offspring still needs to be characterized.

Only a few studies have addressed the natural vertical transmission of Zika virus in wild mosquito populations; most of these have been carried out in Brazil, where Zika virus RNA has been detected in male *Ae. aegypti* mosquitoes and in adult *Ae. albopictus* mosquitoes raised from field-collected eggs (*26*,*27*).

In this study, we demonstrated the occurrence of natural vertical transmission of Zika virus in wild mosquito populations from the municipality of Jojutla in the state of Morelos, Mexico. The RNA of Zika virus was detected in 17 larvae pools; the rates of vertical transmission of Zika virus in the wild *Ae. aegypti* populations were estimated by calculating the MIR. In Morelos, the rainy season begins in late May and extends through the end of September, after which both the precipitation and the mosquito populations start to decrease. Thus, the higher MIR (6.95) calculated from the larvae hatched from the eggs collected in November 2016, in contrast to the MIR from the larvae hatched from the eggs collected in June (2.6), might be correlated with the increased number of human Zika virus cases that were reported in Jojutla during November, whereas during June no human cases were reported. The absence of reported Zika virus human cases in June could be the result of asymptomatic cases, cases clinically misdiagnosed as dengue virus infections, or both.

The higher number of persons infected with Zika virus in Jojutla during November reflects an increase in the number of infected mosquitoes resulting from horizontal transmission of the virus between mosquitoes and infected humans. It is possible that vertical transmission is contributing to the number of infected mosquitoes, which are, in turn, capable of transmitting this virus to a higher number of humans. However, the relative importance of vertical transmission for the maintenance and spread of the virus cannot be elucidated with the data available so far.

The MIR is usually affected by the number of mosquitoes that make up the pool of mosquitoes (or their immature stages) tested, which usually causes an underestimation of the real number of infected mosquitoes in a population (*10*). Thus, the rate of vertical transmission in the mosquito populations from Morelos might be even higher than estimated. Previous studies performed with other flaviviruses, such as DENV, have reported MIRs as low as 0.18 (*28*) and 0.6 (*29*) or as high as 40 (*30*) in wild larvae, usually associated with the collection date and the place of sampling. Under laboratory conditions, the filial infectious rate of Zika virus in *Ae. aegypti* mosquitoes ranged from 4.9 to 24.6, depending on the strain of the virus tested (*31*), which corroborates that our results are comparable to the MIRs reported for other Zika viruses and other flaviviruses, including DENV (*32*,*33*).

In this work, we were also able to demonstrate the natural vertical transmission of Zika virus in *Ae. aegypti* mosquitoes by the successful isolation of infectious Zika virus (31N) from larvae raised from field-collected eggs. This evidence strongly suggests that infectious Zika virus can be transmitted from adult female mosquitoes to their offspring and increases the evidence of the role of natural vertical transmission in the maintenance of Zika virus in wild mosquito populations.

The isolation of infective Zika virus vertically transmitted from adult mosquitoes to their offspring suggests that this virus could be potentially transmissible between mosquitoes and their vertebrate hosts; nevertheless, this transmission still needs to be demonstrated. Moreover, we were able to detect several SNVs in the larvae-derived Zika virus isolate 31N, of which 3 were specific to this larvae-derived genome, as well as 10 others that were shared between the larvae and other mosquito and human sequences. Although it is plausible that these SNVs were acquired during virus culture, we think this is unlikely because 72 of the sequences included in the alignment corresponded to cultured viruses and none of them presented these mutations; however, the sequences from more larvae-derived viruses need to be determined to establish the significance of these SNVs. The larvae-specific SNVs were located in the coding regions for the NS2A and NS3 proteins, which are involved in the replication of the viral RNA (*34*). Nevertheless, whether the larvae-specific SNVs are associated with the maintenance and vertical transmission of infectious Zika virus from adult mosquitoes to their offspring still needs to be determined.

In summary, we demonstrated natural vertical transmission of Zika virus in wild *Ae. aegypti* mosquitoes. Our results suggest that this transmission mode could aid in the spread and maintenance of Zika virus in nature, expanding the ongoing zoonotic threat from this virus to human health.
